# Editorial on Special Issue: Computational Insights into Calcium Signaling

**DOI:** 10.3390/biom15040485

**Published:** 2025-03-26

**Authors:** Mohsin Saleet Jafri, Aman Ullah

**Affiliations:** 1 School of Systems Biology, George Mason University, Fairfax, VA 22030, USA; aullah3@gmu.edu; 2 Center for Biomedical Engineering and Technology, University of Maryland School of Medicine, Baltimore, MD 20201, USA

Calcium is a ubiquitous second messenger and plays a major role in a variety of cellular functions, both within the same cell and between different cells [1,2]. These functions include contraction, secretion, cellular transport, fertilization, gene expression, metabolism, disease pathology, and others. Calcium is able to control different processes due to its tight regulation, both temporally and spatially. In disease, calcium signaling has often been altered and can underlie the cellular changes behind the disease phenotype.

Computational approaches such as modeling and simulation, informatics analysis, molecular simulation, machine learning, signal processing, image analysis, and others have helped scientists gain insights into the complexity of calcium signaling. This Special Issue of Biomolecules entitled “Computational Insights into Calcium Signaling” highlights how computational approaches have been used to understand the mechanisms and consequences of calcium signaling. Contributions in the form of original experimental articles, up-to-date reviews, or short communications are welcome. 

This Special Issue consists of six papers: four original research papers and two review papers. Three of these papers have been chosen as “Editor’s Choice” by the scientific editors of MDPI. The common theme of these papers is that they explore the role of calcium signaling in mammalian cells in a normal or disease state to understand the mechanisms of these processes using computational models. 

Calcium signaling plays a major role in heart function. During depolarization of the cardiac ventricular myocyte, voltage-gated L-type Ca^2+^ channels open, allowing Ca^2+^ entry into the cell. This Ca^2+^ influx into the dyadic subspace activated Ca^2+^-dependent Ca^2+^ release from the ryanodine receptors, thereby releasing Ca^2+^ from the sarcoplasmic reticulum known as a calcium-induced calcium release (CICR) phenomenon. This 10–20-fold increase in myoplasmic [Ca^2+^] triggers contraction by binding to the myofilaments. The amount of Ca^2+^ entry and amplification will vary depending upon the pacing frequency. Dysregulation of this calcium signaling system during disease can lead to cardiac arrhythmia and contractile dysfunction.

In the Editor’s choice paper “The Role of Ca^2+^ Sparks in Force Frequency Relationships in Guinea Pig Ventricular Myocytes” Paudel et al. (contribution 1), develops a model for calcium signaling and the membrane currents for the Guinea pig ventricular myocyte that can show the effects of the local calcium signaling, Ca^2+^ sparks. The force frequency relationship in Guinea pig ventricular myocytes show a rising and then falling curve for force generated against pacing frequency. With the model, they demonstrate that diastolic sarcoplasmic reticulum [Ca^2+^] and adaptation of the ryanodine receptor both increase with stimulation frequency, producing this rising then falling amplitude of myoplasmic [Ca^2+^] transients. Furthermore, the reduction in the L-type Ca^2+^ current, with an increase in pacing frequency due to Ca^2+^-dependent inactivation, also plays a role in the negative slope of the force–frequency relationship.

In the research Editor’s choice paper “Local Control Model of a Human Ventricular Myocyte: An Exploration of Frequency-Dependent Changes and Calcium Sparks”Alvarez et al (contribution 2). develops a computational model for the human ventricular myocyte to perform a deeper exploration of how local calcium signaling in the form of Ca^2+^ sparks leads to force frequency dynamics in the human heart. In addition to studying force frequency similar to the Paudel et al. paper, they dissected the mechanisms of action potential and mechanical restitution of the myocyte, learning that the Ca^2+^-dependent activation rate show that the duration of LCC opening helps modulate its effects on the APD restitution at different diastolic intervals.

A review paper in this Special Issue “Modeling Calcium Cycling in the Heart: Progress, Pitfalls, and Challenges” written by Qu et al (contribution 3). also addresses calcium signaling in the heart. They explain the different types of models used to represent the ryanodine receptor and how different modeling strategies can be used to represent the dyadic arrangements that are responsible for the local calcium signaling. Cardiac myocytes are arranged in an electrically coupled network in the heart, and the paper describes how models have been used to represent this. The review also describes the role of mitochondria in calcium signaling dynamics, which leads to the next paper in this Special Issue.

The review paper “Calcium Overload and Mitochondrial Metabolism” by Walkon et al. (contribution 4), offers a different topic of calcium signaling, and describes how Ca^2+^ is important to regulate the function of the mitochondrial by activating both the tricarboxylic acid cycle and the electron transfer chain to boost ATP, the energy currency of the cell, when calcium is elevated as is the case when the heart rate increases or during neuron activity. They also explain that during the pathological condition of calcium overload that can occur during disease, mitochondrial [Ca^2+^] can be detrimental to mitochondrial function.

The Editor’s choice research paper “Synaptic Plasticity Is Predicted by Spatiotemporal Firing Rate Patterns and Robust to In Vivo-like Variability” written by Dorman and Blackwell (contribution 5) studies calcium signaling in neurons and its contribution to neural synaptic plasticity. Synaptic plasticity is the activity-dependent modification of synaptic strength. The authors investigate how the spatiotemporal synaptic input patterns can produce plasticity. They do so by using in vivo-like conditions using a data-driven computational model with a plasticity rule based on calcium dynamics to find that the inputs control the direction and magnitude of the plasticity. The strongly potentiated synapses have greater firing rates and [Ca^2+^] compared to depressed synapses.

In salivary gland acinar cells, agonist stimulation leads to the production of inositol 1,4,5-trisphosphate (IP_3_), which mobilized calcium from the endoplasmic reticulum via the IP_3_ receptor. The ensuing rise in [Ca^2+^] activates pumps that move water out of the cell. In the research paper “Simulation of Calcium Dynamics in Realistic Three-Dimensional Domains” Sneyd et al (contribution 6). studies the spatial distribution of Ca^2+^ in salivary gland acinar cells to explore how this influences saliva secretion, which depends on [Ca^2+^]. They describe the importance of capturing realistic anatomical details of the salivary gland acinar cells as crucial to producing realistic calcium signaling and secretion dynamics. Because this process is time-consuming and very sensitive to error, the authors present an automated approach they have developed to address these challenges.

In conclusion, calcium signaling plays a role in many different cell types for different purposes. This Special Issue captures a few examples of the importance of calcium signaling and how computational models have helped to clarify the roles and mechanisms of calcium signaling.

## Abbreviations

The following abbreviations are used in this manuscript:

Ca^2+^	Calcium ions
[Ca^2+^]	Calcium ion concentration
ATP	Adenosine triphosphate
IP_3_	Inositol 1,4,5-trisphosphate

## References

[1] Bootman M.D., Bultynck G. (2020). Fundamentals of Cellular Calcium Signaling: A Primer. Cold Spring Harb. Perspect. Biol..

[2] Berridge M.J., Bootman M.D., Roderick H.L. (2003). Calcium signalling: Dynamics, homeostasis and remodelling. Nat. Rev. Mol. Cell Biol..

